# Advanced Squamous Cell Carcinomas of the Pelvic and Perineal Region: A Comprehensive Genomic Profiling Study

**DOI:** 10.1093/oncolo/oyac144

**Published:** 2022-07-26

**Authors:** Andrea Necchi, Philippe E Spiess, Marco Bandini, Giuseppe Basile, Petros Grivas, Gennady Bratslavsky, Joseph Jacob, Natalie Danziger, Douglas Lin, Brennan Decker, Ethan S Sokol, Richard S P Huang, Sanjay B Kulkarni, Jeffrey S Ross

**Affiliations:** IRCCS San Raffaele Hospital and Scientific Institute, Milan, Italy; Vita-Salute San Raffaele University, Milan, Italy; Moffitt Cancer Center and Research Institute, Tampa, FL, USA; IRCCS San Raffaele Hospital and Scientific Institute, Milan, Italy; Vita-Salute San Raffaele University, Milan, Italy; IRCCS San Raffaele Hospital and Scientific Institute, Milan, Italy; Vita-Salute San Raffaele University, Milan, Italy; University of Washington, Fred Hutchinson Cancer Center, Seattle, WA, USA; SUNY Upstate Medical University, Syracuse, NY, USA; SUNY Upstate Medical University, Syracuse, NY, USA; Foundation Medicine, Inc., Cambridge, MA, USA; Foundation Medicine, Inc., Cambridge, MA, USA; Foundation Medicine, Inc., Cambridge, MA, USA; Foundation Medicine, Inc., Cambridge, MA, USA; Foundation Medicine, Inc., Cambridge, MA, USA; Kulkarni Reconstructive Urology Center, Pune, Maharashtra, India; Foundation Medicine, Inc., Cambridge, MA, USA; SUNY Upstate Medical University, Syracuse, NY, USA

**Keywords:** comprehensive genomic profiling, pelvic cancer, squamous cell carcinoma, biomarkers, targeted therapy, immunotherapy

## Abstract

**Background:**

Advanced pelvic squamous cell carcinoma (pSCC) is a broad category of cancers affecting different pelvic organs and usually featuring unfavorable clinical outcomes. Thus, we aimed to assess genomic differences among pSCC cases and learn whether pSCC could potentially benefit from targeted therapies and/or immunotherapy.

**Materials and Methods:**

A total of 1917 advanced pSCCs, including penile (penSCC), male urethral (murthSCC), male anal (manSCC), female urethral (furthSCC), vulvar (vulSCC), cervical (crvSCC), female anal (fanSCC), and vaginal (vagSCC), underwent comprehensive genomic profiling (CGP). We used hybrid capture-based CGP to evaluate recurrent genomic alterations (GAs). Tumor mutational burden (TMB) was determined on up to 1.1 Mb of sequenced DNA and microsatellite instability (MSI) was determined on up to 95 loci. Programmed cell-death-ligand-1 (PD-L1) expression was determined by immunohistochemistry (IHC; Dako 22C3).

**Results:**

*PIK3CA* was the most frequently identified potentially “actionable” GA (22%-43%), followed by *mTOR* pathway [*PTEN* (0%-18%), *FBXW7* (7%-29%)], and cell-cycle GAs. DNA-damage response (DDR) GAs and receptor-tyrosine kinase (RTK) targeted options were uncommon. *NOTCH1* GAs were present in >15% of penSCC and vulvSCC. TMB ≥10 mut/Mb was >15% in manSCC, fanSCC, crvSCC, and vagSCC. PD-L1 high expression was >18% in all pSCC except urthSCC, manSCC, and vagSCC. HPV-16/18 detection was highest in manSCC, fanSCC, and crvSCC.

**Conclusion:**

Despite similar histology, pSCCs can differ in GAs and HPV status. Overall, *PIK3CA* is the most frequent potentially “targetable” GA followed by *mTOR* and cell cycle pathway. RTK and DDR GAs are rare in pSCC. Immunotherapy could be considered for pSCC management based on TMB and PD-L1 expression.

## Teaser

This study assesses genomic differences among a large series of pelvic squamous cell carcinoma (pSCC) cases to determine whether pSCC could potentially benefit from targeted therapies and/or immunotherapy.

**Implications for Practice**

Squamous cell carcinomas of the pelvic and perineal region include a variety of rare tumors, most of which suffer from limitations in accessing clinical trial options or new drug development for cases developing resistance to conventional treatments. In order to bridge this knowledge gap and allow clinicians to consider the availability of druggable targets in these rare tumors, this manuscript presents the data from the largest series of such cases. From these data, a wide discrepancy among independent tumors emerges, providing a rationale for the consideration of this group of malignancies for enrollment in basket or umbrella studies testing new drugs.

## Introduction

Squamous cell carcinoma (SCC) can arise from any male and female pelvic or perineal surfaces including penile, urethra, anus, vulva, vagina, and cervix. In most cases, pelvic or perineal SCC (pSCC) represents a rare entity accounting for less than 1% of all tumors in both genders^1^; the only exception being the uterine cervix SCC, which is a high prevalent disease especially in developing countries where human papillomavirus (HPV) infection is endemic despite the vaccine development.^2^ Clinical manifestations, disease course, and treatment options are widely different among pSCCs. However, while surgery and chemoradiation have improved survival for localized disease, patients with advanced pSCC have unfavorable outcomes regardless of treatment.^3-6^ Furthermore, systemic treatment options are limited for advanced pSCC and surgery typically leads to significant anatomic defects, with subsequent sexual and functional impairment.

Although pSCCs share similar histological features and potential drivers of malignancy, including HPV infection,^7^ little is known about the genomic profiles of these tumors compared with those SCC that are localized outside the perineum and pelvic area. In this context, important advances have been gained in the management of non-perineal SCC from genomic profile evaluation. Particularly, following the results of Bonner et al study^8^ for locally advanced head and neck SCC (HNSCC) and the EXTREME study^9^ for recurrent/metastatic HNSCC, cetuximab was approved as the first targeted drug for HNSCC. Similarly, encouraging results were achieved from phase III trials testing anti-programmed cell-death-1/ligand-1 (PD-1/L1) immune-checkpoint inhibitors (ICIs) nivolumab^10^ and pembrolizumab,^11^ showing overall survival benefit for patients with recurrent/metastatic HNSCC irrespective of PD-L1 expression and HPV status. Quite recently, pembrolizumab was approved by the US Food and Drug Administration (FDA) in combination with chemotherapy, with or without bevacizumab, for the treatment of persistent, recurrent, or metastatic cervical carcinoma, including cases with squamous cell histology.^12^ To date, however, no targeted therapies have been approved for pSCC, where the current approach remains largely limited to chemoradiotherapy.^13-15^ Thus, considering the rarity of pSCCs and the very aggressive course of advanced disease, there is a critical need to improve our understanding of the molecular underpinnings of pSCC to provide insights that could impact future treatment options. Here, we present results from a comprehensive genomic profiling (CGP) of advanced pSCCs focused on the identification of recurrent and currently potentially targetable genomic alterations (GAs), including those co-segregating with HPV status.

## Materials and Methods

This study was approved by the Western Institutional Review Board (Protocol No. 20152817) which issued an approval waiver of informed consent and a HIPAA waiver of authorization. DNA was extracted from either paraffin tissue blocks or a minimum of 40-µm thick unstained tissue section on glass slides on 1917 advanced pSCCs. Using the submitted pathology reports and corresponding relevant clinical information, the tissue samples were classified as either obtained from metastatic site biopsies or from sites of unresectable loco-regional disease. CGP was performed with FoundationOne CDx next-generation sequencing in a Clinical Laboratory Improvement Amendments (CLIA)-certified, College of American Pathologists (CAP)-accredited laboratory as previously described.^16^ Particularly, all cases included in this study were evaluated by an experienced board-certified pathologist at the time of specimen arrival in the laboratory and then reviewed by a single pathologist to confirm the diagnosis. Sections were macro dissected to achieve ≥ 20% estimated percent tumor nuclei (TN) cellularity, defined as the number of tumor cells divided by the total number of nucleated cells as assessed by light microscopy. Next, ≥55 ng DNA were extracted from formalin-fixed, paraffin-embedded (FFPE) tumor samples. Samples were assayed by adaptor ligation hybrid capture, performed for all coding exons of 309 cancer-related genes plus select introns from 34 genes. Sequencing was performed using the Illumina HiSeq instrument to a median exon coverage ≥ 500×, and data were analyzed for all classes of GAs. The computational pipeline used to analyze sequence patterns used Bayesian algorithms to identify base substitution mutations, local assembly to identify short insertions and deletions, comparisons with process matched normal controls to determine gene amplifications and homozygous deletions and the analysis of chimeric read pairs to identify gene re-arrangements and gene fusions.^16^ The test was previously optimized and validated to detect base substitutions at a ≥ 5% mutant allele frequency (MAF), indels with a ≥ 10% MAF with ≥ 99% accuracy, and fusions occurring within baited introns/exons with > 99% sensitivity to maximize mutation-detection accuracy.^16^ The prediction of germline status for this study used the extracted somatic DNA and was performed using a computational approach to distinguish somatic vs germline origin of GAs.^17^ Microsatellite instability (MSI) status was determined on up to 95 loci.^18,19^ Using 0.8 to 1.1 Mb of sequenced DNA for each case, the tumor mutational burden (TMB) was determined using the number of somatic base substitution or indel alterations per Mb after filtering to remove germline and pathogenic mutations.^20^ The DAKO 22C3 antibody was used to determine PD-L1 tumor cell expression using 5-µm tissue sections. Following the current practice for non–small cell lung carcinomas, PD-L1 expression was determined on subsets of the tumors using the DAKO 22C3 assay with low positive tumor cell scoring defined as 1%-49% staining and high positive tumor cell scoring defined as ≥ 50% staining.

In this manuscript, the attribution of the definition of “targetable” to any single gene or genomic pathways is based on the current knowledge of available therapeutic compounds already approved in any tumor types. Such a definition relied on an informal consensus among authors, based on the review of existing approved testing and therapy combinations, similar to what has been previously reported by the American Society of Clinical Oncology (ASCO)^21^ and the European Society for Medical Oncology (ESMO),^22^ added to emerging clinical trial data.

Analyses were descriptive in nature and no formal statistical tests were applied to compare the frequencies between subgroups.

## Results

CGP was performed on 230 penile (penSCC), 17 male urethral (murthSCC), 125 male anal (manSCC), 7 female urethral (furthSCC), 263 vulvar (vulSCC), 822 cervical (crvSCC), 277 female anal SCCs (fanSCC), and 176 vaginal (vagSCC) tumor samples.

The samples used for sequencing in the pSCC cases were a mix of both primary site tissues (52%-60%) and regional and metastatic site tissues (40%-48%).

**Figure 1.**
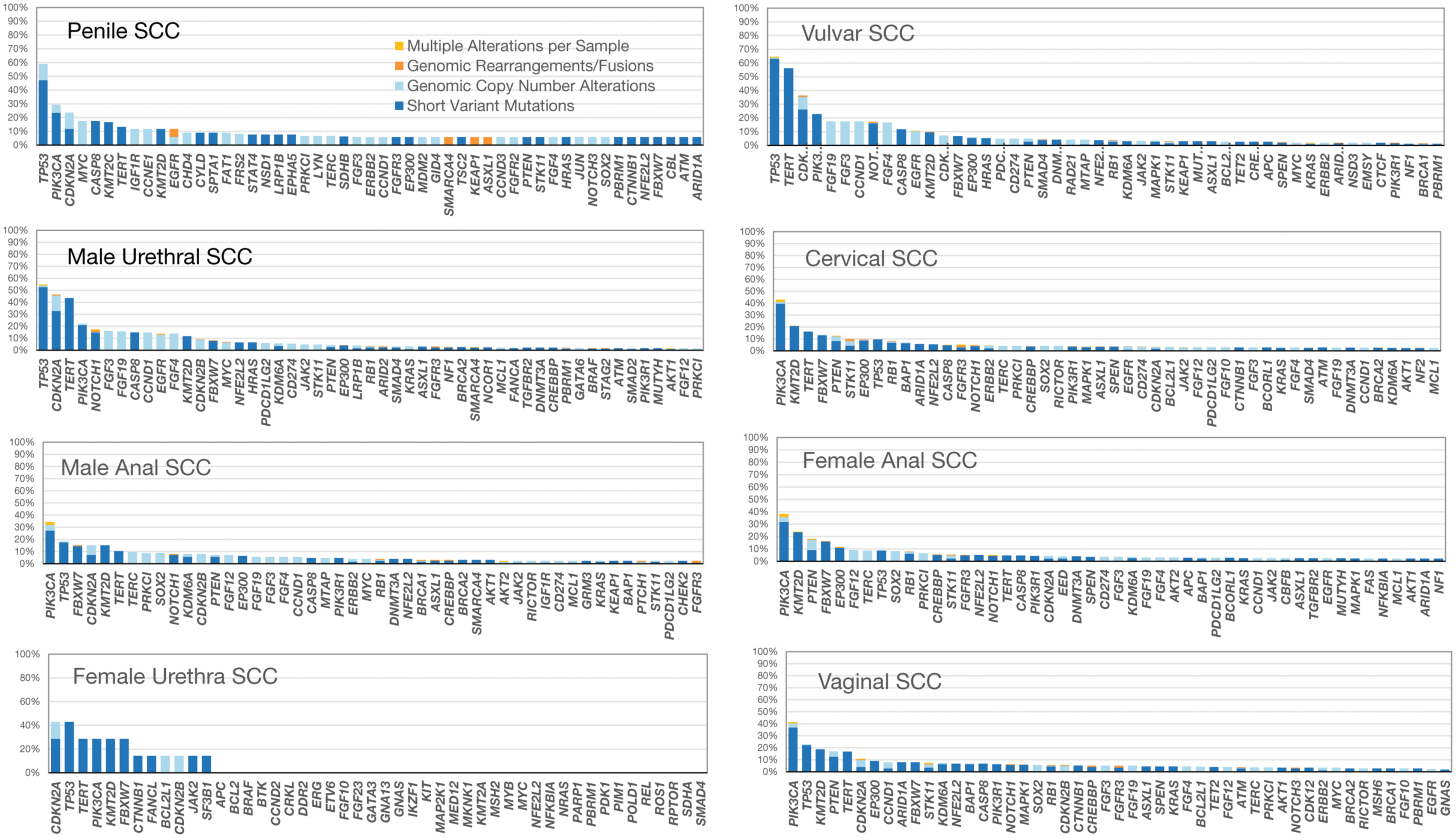
Longtail plots of genomic alterations in pelvic and perineal squamous cell carcinomas. Abbreviation: SCC squamous cell carcinoma.

Clinical features, HPV status, mean number of GA per tumor, a selected set of GAs and biomarkers associated with immunotherapy response for the eight subtypes of pSCC are reported in Table 1. Longtail plots of the GA in each tumor type are presented in Fig. 1. Women with crvSCC were younger than men and women with other pSCC. HPV-16/18 detection was lowest in murthSCC (12%), vulSCC (25%), and penSCC (29%) and highest in manSCC (73%), fanSCC (90%), and crvSCC (68%) and *TP53* GAs appeared inversely correlated with high-risk HPV status.

**Table 1.** Frequency of genomic alterations in 1917 cases of pelvic or perineal squamous cell carcinomas in men and women.

	Penile SCC (*n* = 230)	Male Urethral SCC (*n* = 17)	Male Anal SCC (*n* = 125)	Female Urethral SCC (*n* = 7)	Vulvar SCC (*n* = 263)	Cervical SCC (*n* = 822)	Female Anal SCC (*n* = 277)	Vaginal SCC (*n* = 176)
Median age (range), years	65 (24-92)	63 (40-76)	60 (26-89+)	61 (49-75)	64 (29-89+)	51 (22-89+)	62 (35-89+)	62 (29-89+)
GAs/tumor , %	5.7	4.9	4.2	3.6	5.4	4.4	4.1	5.1
HPV-6/11 (low risk), %	3	0	6	0	1	<1	1	0
HPV-16/18 (high risk), %	29	12	73	43	25	68	90	47
Currently untargetable genomic alterations, %								
*TP53*	55	59	18	43	65	10	9	23
*TERT* promoter mutation	44	13	10	29	56	16	5	17
*CCND1* amplification	15	6	6	0	18	3	3	8
*CDKN2A* Loss	47	24	15	43	37	4	4	11
*CDKN2B* Loss	9	0	8	14	7	2	2	6
Currently targetable genomic alterations in other tumor types, %								
*PIK3CA*	22	30	34	29	23	43	38	42
*PTEN* inactivation	4	6	7	0	5	13	18	17
*BRCA1*	<1	0	3	0	2	1	2	3
*BRCA2*	3	0	3	0	2	3	1	3
*EGFR* amplification	14	12	1	0	10	3	2	2
*FGFR3*	3	6	2	0	1	5	5	5
Potential targetable genomic alterations in clinical development, %								
*NOTCH1*	17	0	8	0	17	5	5	0
*MTAP* Loss	6	22	5	0	3	1	2	<1
*FBXW7*	8	6	15	29	7	14	16	8
Immunotherapy response-associated biomarkers, %								
*CD274* amplification	6	0	2	0	5	4	4	2
MSI high	1	0	<1	0	<1	1	1	3
Median TMB	3.8	3.8	5.0	3.8	3.8	5.0	5.0	5.0
Mean TMB	5.7	6.5	7.17	3.6	5.7	8.1	7.0	9.2
TMB >10 mt/Mb	15	6	24	0	11	27	22	28
TMB >20 mt/Mb	5	6	5	0	3	9	5	15
PD-L1 low positive (1%-49%), *n/N* (%)	32/128 (25)	3/11 (28)	30/60 (50)	3/4 (75)	61/143 (43)	2/22 (9)	54/112 (48)	35/70 (50)
PD-L1 high positive (≥50%), *n/N* (%)	44/128 (34)	2/11 (18)	11/60 (18)	0/4 (0)	47/143 (33)	6/22 (27)	25/112 (22)	11/70 (16)

Abbreviations: GA, genomic alterations; HPV, human papillomavirus; MSI, microsatellite instability; PD-(L)1, programmed cell-death (ligand)-1; SCC, squamous cell carcinoma; TMB, tumor mutational burden.

GAs in *PIK3CA* were the most frequent potentially targetable biomarkers with crvSCC (43%), vagSCC (42%), and fanSCC (38%) harboring the highest GAs rate, and penSCC (22%) and vulSCC (23%) the lowest. *PTEN* inactivating mutations and deletions were more frequent in female pSCC, including crvSCC, fanSCC, and vagSCC which ranged from frequencies of 13% to 18%. In addition to these m*TOR* pathway alterations, the *FBXW7* GA frequencies, putative targets for novel *mTOR* inhibitors were similarly higher in females with crvSCC, vulSCC, and vagSCC and highest in the furthSCC cohort at 29%.

DNA-damage response (DDR) GAs (eg, *BRCA1/2*, *ATM*, *ATX*, *ATR*, *ERCC2*, *ERCC4*, *FANCC*) were very low (<1%-3%) throughout the cohort, with germline *BRCA* GA extremely rare (0%-3%). Similarly, receptor-tyrosine kinase (RTK) targeted options were quite uncommon as only 1% GA frequencies in *BRAF*/*ERBB2* were identified. Although *EGFR* GA were noteworthy in the penSCC (14%) and murthSCC (12%) types, most of the GAs were *EGFR* copy number increases, while activating *EGFR* short variant mutations were extremely rare. For the uncommon *EGFR* GAs found in the pSCC cases, the overwhelming majority were copy number changes (amplifications) with far <1% of all pSCC harboring either activating point mutations or gene fusions. The *FGFR3* GA were also extremely uncommon (0%-6%) and featured a mix of amplification, activating short variant mutations and gene rearrangements. A series of currently “non-targetable” GA via available drugs were widely distributed in the pSCC groups. *TERT* promoter GA were most frequent in the cutaneous pSCC tumors, including penSCC (44%) and vulSCC (56%). GA associated with cell cycle dysregulation were also widely distributed. Similar to *TERT* promoter GA frequencies, *CCND1* amplification was most frequently detected in the penSCC (15%) and vulSCC (18%) tumor. *CDKN2A* loss was more widely distributed across pSCC subtypes, excluding crySCC (4%) and fanSCC (4%) types.

**Figure 2.**
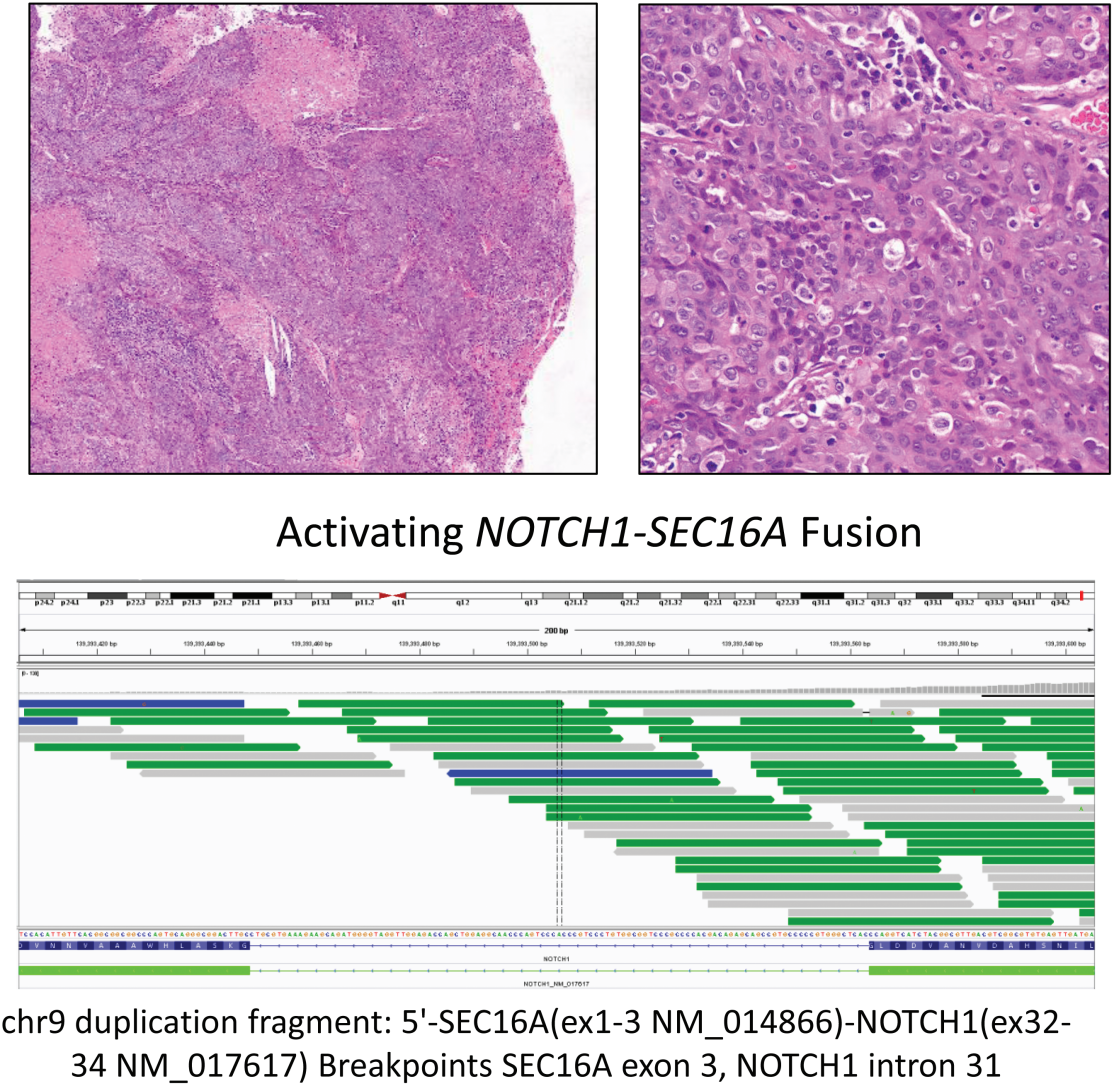
Low (5×) (upper left) and high (20×) (upper right) magnification of a large cell non-keratinizing SCC of the cervix in a 75-year-old woman which progressed to stage IV disease (Hematoxylin and Eosin). On comprehensive genomic profiling this tumor was found to contain HPV-18 DNA, was MS stable and had a TMB of 11 mutations/Mb. This tumor featured a T1025fs*23 somatic BRCA1 frameshift short variant mutations and a NOTCH1 fusion (low center) which is predicted activate the NOTCH1 pathway. In addition to potential use of immunotherapy for the TMB > 10 mut/Mb and PARP inhibitors for the BRCA1 inactivating mutation, this patient would be eligible for clinical trials featuring NOTCH1 inhibitors, γ-secretase inhibitors and anti-NOTCH antibody therapeutics.

Finally, when considering other potential targetable GAs, *NOTCH1* GAs frequencies were present in >15% of penSCC and vulvSCC. A case example of a crySCC driven by NOTCH pathway dysregulation is shown in Fig. 2. Conversely, *MTAP* loss was notably more frequent in male murthSCC (22%) than in the other pSCC cases ranging from <1% to 6%.

**Figure 3.**
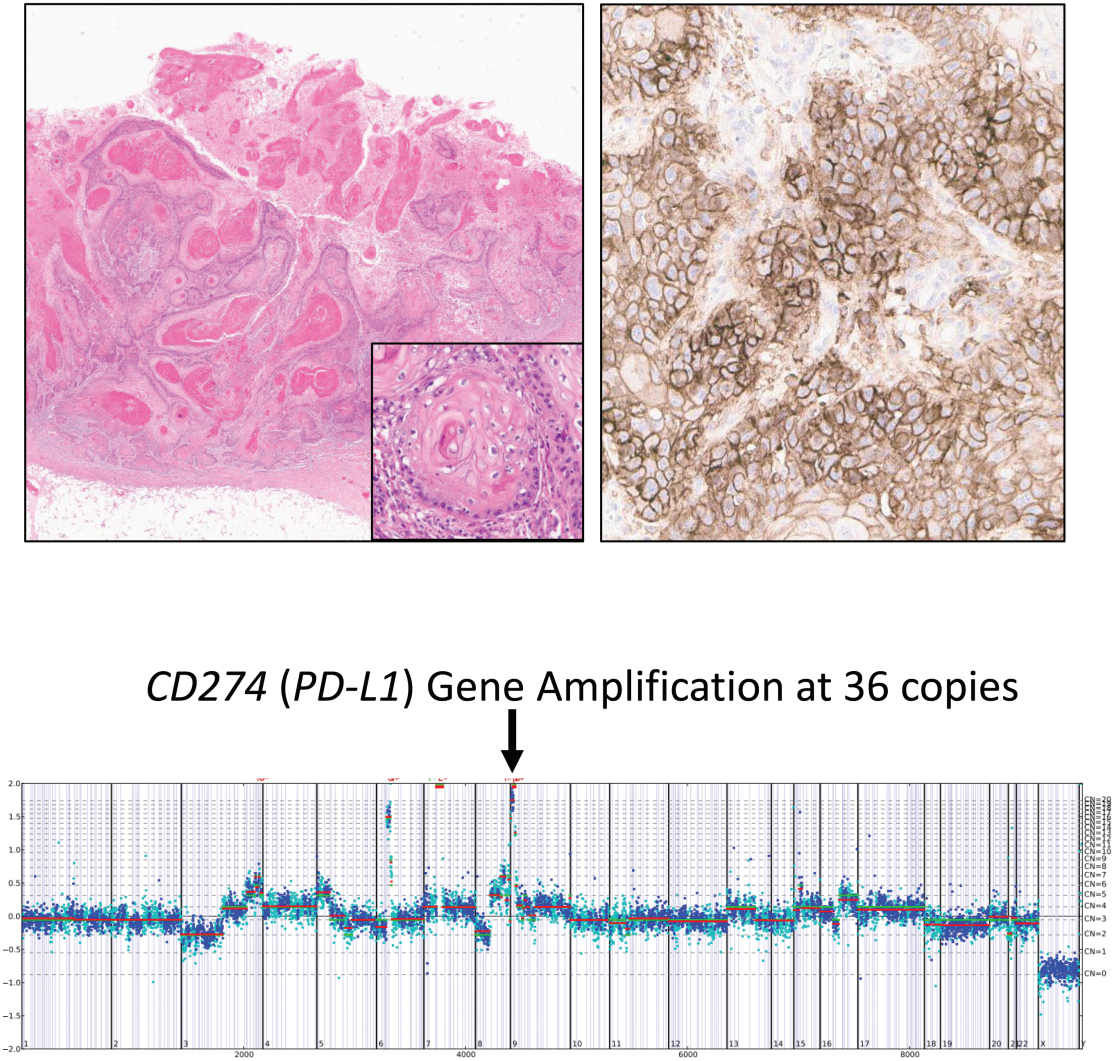
Penile SCC metastasis in a lymph node in a 55-year-old man. Upper left shows a hematoxylin and eosin-stained low magnification image (5×) of the primary tumor with a high magnification detail shown in the upper right (20×). This tumor was HPV negative, was MS-stable and had a low TMB of 3 mutations/Mb. Comprehensive genomic profiling revealed major amplification of the PD-L1 gene (CD-274) at 36 copies/cell which is shown in the copy number plot in the low center. The image on the upper right shows 100% membranous tumor cell staining for PD-L1 expression using the DAKO 22C3 anti-PD-L1 antibody. This tumor also featured mutations in *CDKN2A*, *NFE2L2*, *TERT*, and *TP53*. Other amplified genes included *CCND3*, *EGFR*, *JAK2*, *PD-L2*, and *VEGFA*. In summary, although immunotherapy might not have been considered given the MSI and TMB status, the PD-L1 amplification and overexpression are potential strong indicators of efficacy of immune-checkpoint inhibitors.

The list of biomarkers possibly associated with ICI efficacy are also included in Table 1. The frequencies of *PD-L1* (*CD274*) gene amplification had low levels in murthSCC, manSCC, femurthSCC, and vagSCC (0-2%). Conversely, vulSCC, crvSCC and fanSCC and penSCC reached higher level (4-6%). A case example of a metastatic penSCC with major amplification of *CD-274* is shown in Fig. 3. MSI-High status was extremely uncommon in pSCC with most groups featuring only 0-1% frequencies apart from the vagSCC type reaching 3%. The highest frequencies of TMB ≥ 10 mutations/Mb, an FDA-approved biomarker for identifying pembrolizumab eligibility in a pan-cancer setting, were found in the penSCC (15%), manSCC (24%), crvSCC (27%), fanSCC (22%), and vagSCC (28%). PD-L1 low expression prevalence was > 25% in all pSCC except crvSCC and high expression prevalence was > 18% in all pSCC except male and female urthSCC, manSCC, and vagSCC with the penSCC, vulSCC, and crvSCC showing the highest frequencies of PD-L1 high-level expression.

When the individual tumor types are summarized independently for targetable GAs, highlighted observations include a modest high-risk HPV incidence in penSCC when compared with crvSCC; higher frequencies of *EGFR* amplification in penSCC, murthSCC, and vulSC; higher rates of NOTCH pathway alterations in penSCC and vulSC; and the highest incidence of *MTAP* loss in the murthSCC cases. Analysis of GAs and ICI efficacy and resistance biomarkers did not uncover novel co-occurrences. Previously described co-occurrences including the *CCND1*/*FGF3*, *4*, *19* amplicon, *CDKN2A/B* co-deletions occasionally with *MTAP* deletion were routinely identified as was the association of *PD-L1* (*CD274*) amplification with PD-L1 expression by immunohistochemistry (IHC). For the *FBXW7* gene, when altered in the entire cohort of pSCC, other MTOR pathway alterations were also identified including *PIK3CA* (45.4% of *FBXW7* altered pSCC) and *PTEN* (13.9% of *FBXW7* altered pSCC). In addition, for the entire pSCC cohort that featured *PIK3CA* GAs, *PTEN* was co-altered in 10.2% of cases and, for the entire cohort that featured *PTEN* alterations, 30.3% of the cases also had *PIK3CA* GAs.

## Discussion

Identifying actionable GAs is a critical step for the development of precision and personalized oncology and to offer potential novel therapeutic options. However, while the management of high prevalent tumors has been revolutionized by identification of potential molecular targets,^23^ treatment options for rarer cancer types remain disappointingly limited. Indeed, little is known about the genomic landscape of rare tumors leaving uncertainty as to whether targeted therapies would also be effective in these malignancies. Moreover, given the rarity of these tumor types, few genomic investigations have been carried to explore possible activation of potentially “targetable” pathways.^24-26^

In our cohort of pSCCs, sequence alterations in *PIK3CA* were the most commonly targetable GAs noted across the entire population with crvSCC, vagSCC, and fanSCC harboring the highest frequencies of GAs, and penSCC and vulSCC the lowest. Preclinical models reported that the *PI3K/AKT/mTOR* pathway is frequently activated in human cancers,^27^ and that its activation could lead to therapy resistance.^28^ To date, the strongest results of *PI3KCA* pathway inhibition have been achieved in metastatic breast cancer with activating *PIK3CA* mutations treated with the FDA-approved *PIK3CA* inhibitor alpelisib or the *AKT* inhibitor ipatasertib.^29,30^ Thus, following these encouraging results, *PI3K/AKT/mTOR* inhibitors are currently investigated as monotherapy or in combination in different tumors harboring activating mutations.^31^ Indeed, we should acknowledge the limitations that historically characterized the efforts of identifying those patients who are most suited for such targeted therapies. Until recently, results from clinical trials with PI3K inhibitors in solid tumors have been largely disappointing.^32^

Additionally, *PI3K/AKT/mTOR* pathway hyperactivation could results from other molecular abnormalities different from *PIK3CA* mutation or amplification, such as *PTEN* loss-of-function or *FBXW7* mutation, as also identified in our analysis. *PTEN* inactivating mutations and deletions were frequent in our study, especially in female pSCCs including crvSCC, fanSCC, and vagSCC, while *FBXW7* frequencies were higher in female gender patients with crvSCC, vulSCC, and vagSCC and especially in furthSCC. Particularly, the tumor suppressor gene *PTEN* negatively regulates the *PI3K/AKT/mTOR* pathway,^33^ therefore, *PTEN* deficient tumors could potentially benefit from inhibiting *AKT* or *mTOR*.

These data become even more relevant when considering the proportion of co-occurring molecular alterations. Similarly, *FBXW7* is a tumor suppressor gene mutated in various human tumors.^34^ Particularly, mTOR turnover is regulated by *FBXW7*-mediated ubiquitination, suggesting that inactivating mutations of *FBXW7* could predict sensitivity to *mTOR* inhibitors.^35^ Nevertheless, feedback mechanisms, like activation of *AKT* after *mTOR* inhibition treatment, have been related to reduced therapy efficacy.^36^ Consequently, identification of complicated mutation profiles might be more important and useful than a single gene mutation analysis, as patients might possibly benefit more from therapy combinations and not from a single specific target therapy. Thus, targeting *PI3K/AKT/mTOR* signaling in pSCC could represent a promising treatment option which should be further evaluated in clinical trials.

Apart from *PI3K/AKT/mTOR* pathway alterations, other GAs associated with clinical responses to available targeted therapies were infrequent in our analysis. PARP inhibitors are gaining momentum due to the high response rate in tumors harboring GAs in DNA damage response genes, such as *BRCA1* and *BRCA2* among others.^37^ Nevertheless, GAs linked with DNA damage response genes were uncommon (<1%-3%) throughout our cohort of pSCCs, although there might be a role of PARP inhibitors in relation to DNA damage response gene alterations that are still not fully elucidated yet. Similarly, GAs in RTK genes were infrequent in our cohort. *FGFR3* was mutated in few cases (0%-6%) and most of the GAs associated to *EGFR* were copy number increases, with low presence of activating *EGFR* short variant mutations. Thus, RTK inhibitors therapy may not be that useful in such a cohort of pSCC.

Recently, tumor-specific features have emerged as putative biomarkers associated with the response to ICIs.^38,39^ Of note, it was suggested that a higher frequency of somatic only non-driver gene mutations, denoted as TMB, increase the likelihood of generating immunogenic tumor neoantigens recognized by the host immune system. Nevertheless, the role of TMB as a predictor of immunotherapy response varies significantly by tumor histology and, therefore universal TMB threshold may not be appropriate.^40^ Similarly, high MSI-status can lead to molecular changes linked to ICI responsiveness, possibly via higher TMB and/or abundant tumor-infiltrating lymphocytes. The CGP assay used in this study explored both MSI status and TMB levels. TMB ≥ 10 muts/Mb, a cutoff approved by the FDA for treatment with pembrolizumab,^41^ was common across pSCC variants; however, there were very low numbers of MSI-High status cases, with most groups featuring only 0%-1% frequencies. In addition, high levels of PD-L1 tumor cell expression detected by IHC have been identified only in penSCC, vulSCC, and crvSCC types. Notably, our study revealed that 15% of penSCC and 27%-28% of crySCC, vagSCC, and fanSCC had TMB > 10 mt/Mb, with 15% of vagSCC harboring TMB > 20 mt/Mb. Thus, patients with pSCC should benefit of ICIs treatment based on TMB level, irrespective of a low rate of cases with MSI-high status and high level of PD-L1 expression.

When considering possible differences related to HPV-status, we found that murthSCC (12%) and vulSCC (25%) tumors had the lowest detection of high-risk HPV serotypes, namely 16 and 18, while crvSCC (68%), manSCC (73%), fanSCC (90%) the highest. This difference in HPV-status across pSCC subtypes could be of particular importance, since HPV viral oncoproteins may modulate distinct intracellular signaling pathways, with subsequent effect in tumor development and response to therapy. Indeed, HPV oncoproteins could increase *PI3K/AKT/mTOR* signaling, low concentrations of viral oncoprotein E5 can prevent DNA-damage-induced apoptosis through *EGFR* signaling, while E6 and E7 can also activate *AKT* and *mTOR*37. Of note, *TP53* GAs, not currently targetable by available drugs, appear to be inversely correlated with high-risk HPV status in our analysis.

CGP assay of these pSCC cohort also revealed the presence of several recurrent GAs which are not currently “targetable”. Notably, penSCC (17%) and vulSCC (17%) frequently harbored mutations in the *NOTCH1* pathway that was found to be implicated in cancer development.^42-44^*NOTCH1* inhibition can be achieved through different pathways, among those the most common is through the g-secretase inhibitors, which produce a pan-*NOTCH* inhibition. To date, many preclinical studies evaluated the anti-tumor effect of *NOTCH1* inhibition. However, data are still immature and conflicting leading to a lack of solid position in favor or against the use of *NOTCH1*-targeted therapies in medical oncology.^45^ Nevertheless, our analysis showed that the *NOTCH1* pathway may be an important target in pSCC given is frequent activation and clinical trials of *NOTCH* inhibitors should thus be extended to patients with pSCC.

Finally, our analyses showed a high prevalence of *MTAP* loss in murthSCC (22%), when compared with other pSCC types. Tumors with *MTAP* deletion develop increased levels of intracellular arginine, which renders them susceptible to anti-tumor therapeutic approaches based on a synthetic lethality mechanism. The *MTAP* gene is located adjacent to *CDKN2A* tumor-suppressor gene and is co-deleted with *CDKN2A* in approximately 15% of all cancers.^46,47^ This co-deletion was linked to aggressive tumor behavior and poor prognosis. Multiple clinical trials are currently being conducted which are focused on *PRMT5* or *MTA2* inhibition designed to take advantage of the high intracellular arginine levels in the *MTAP* deficient tumor cells.

In the current study, the greatest limitation is the lack of clinical data annotation, necessary for evaluation of clinical validity and utility of our findings, as well as for assessment of the possible impact of prior therapies on the GA landscape and TMB-level identified. Moreover, the retrospective and descriptive study nature, lack of randomized control groups, the variability in therapies, surveillance programs and follow-up time periods, as well as the presence of possible several selection and confounding factors may impact the interpretation of the results. Nevertheless, results from this pilot comprehensive genomic analysis of advanced pSCC can provide important and relevant insights for continued and accelerated evaluation of potential biomarker-driven trials, assessing the role of targeted therapies for such rare tumors. Also, these data may be useful to provide rationale for inclusion of such rare tumors in basket or umbrella trials testing novel monotherapies or combination therapies, of which some preliminary data have been recently presented.^48,49^

## Data Availability

The data underlying this article will be shared on reasonable request to the corresponding author.
